# Changes in performance over time while learning to use a myoelectric prosthesis

**DOI:** 10.1186/1743-0003-11-16

**Published:** 2014-02-25

**Authors:** Hanneke Bouwsema, Corry K van der Sluis, Raoul M Bongers

**Affiliations:** 1 University of Groningen, University Medical Center Groningen, Center for Human Movement Sciences, UMCG sector F, FA 23, PO Box 196, Groningen NL-9700 AD, The Netherlands; 2 University Medical Center Groningen, Center for Rehabilitation, Groningen, The Netherlands

**Keywords:** Amputee, Grip force control, Kinematics, Motor control, Myoelectric control, Rehabilitation, Skill level, Task performance, Transradial, Upper-limb prosthesis, Visual feedback

## Abstract

**Background:**

Training increases the functional use of an upper limb prosthesis, but little is known about how people learn to use their prosthesis. The aim of this study was to describe the changes in performance with an upper limb myoelectric prosthesis during practice. The results provide a basis to develop an evidence-based training program.

**Methods:**

Thirty-one able-bodied participants took part in an experiment as well as thirty-one age- and gender-matched controls. Participants in the experimental condition, randomly assigned to one of four groups, practiced with a myoelectric simulator for five sessions in a two-weeks period. Group 1 practiced direct grasping, Group 2 practiced indirect grasping, Group 3 practiced fixating, and Group 4 practiced a combination of all three tasks. The Southampton Hand Assessment Procedure (SHAP) was assessed in a pretest, posttest, and two retention tests. Participants in the control condition performed SHAP two times, two weeks apart with no practice in between. Compressible objects were used in the grasping tasks. Changes in end-point kinematics, joint angles, and grip force control, the latter measured by magnitude of object compression, were examined.

**Results:**

The experimental groups improved more on SHAP than the control group. Interestingly, the fixation group improved comparable to the other training groups on the SHAP. Improvement in global position of the prosthesis leveled off after three practice sessions, whereas learning to control grip force required more time. The indirect grasping group had the smallest object compression in the beginning and this did not change over time, whereas the direct grasping and the combination group had a decrease in compression over time. Moreover, the indirect grasping group had the smallest grasping time that did not vary over object rigidity, while for the other two groups the grasping time decreased with an increase in object rigidity.

**Conclusions:**

A training program should spend more time on learning fine control aspects of the prosthetic hand during rehabilitation. Moreover, training should start with the indirect grasping task that has the best performance, which is probably due to the higher amount of useful information available from the sound hand.

## Background

Training programs used nowadays to learn to use an upper limb prosthesis are still clinic specific, [1] rather than evidence-based practice [2,3]. Therefore, it is not known whether a certain training protocol is the most efficient training to facilitate acquisition of prosthetic skills [1]. Hence, there is a growing support for the need of an evidence-based training program [4-7]. To be able to develop such an evidence-based training, knowledge is needed about how people learn to use their prosthesis. Although motor control processes underlying prosthesis use have been examined in a couple of studies [5,7-14], there has been no research to date—to the knowledge of the authors—that studies motor learning processes of goal-directed actions with prostheses over a period of time during multiple practice sessions. This study aims to describe the changes in use of a prosthetic device during practice. The insights of this study can be used to develop an evidence-based training program, and, moreover, might help us understanding underlying motor learning processes.

In general, motor learning is seen as a process that leads to permanent changes in the ability of the learner [15], and is characterized by the changes in performance over time. Although there is no general definition of motor learning, the process is often described by an improvement in the quickness, accuracy, and efficiency of a movement [16-19]. These aspects will therefore form the basis of the outcome measures that will be examined in this study. Next, transfer of performance improvement is investigated in separate testing sessions, as the most important goal of motor learning in rehabilitation is the generalization of the practiced tasks in the clinic to other activities in daily life.

When training an individual, several factors can be addressed to promote the process of motor learning and skill acquisition in general, such as instructions, types of tasks, type of feedback, amount of practice, or the presentation of tasks [20-22]. This study focuses on three aspects that might be important to study when learning to use a prosthesis: i) practice effects over repetitions of individual movements and sessions, ii) the type of tasks practiced, and iii) practice conditions to study grip force control.

The first aspect, effects of practice, is included in the study to capture learning processes over time during multiple practice sessions. This allows us to examine how people learn to use a prosthesis over time. Learning a new skill takes time [23], and, moreover, distributing practice sessions across days instead of only one day of practice—a single day is often the case in motor control learning studies [23]—results in enhanced performance [24]; see [16,25] for studies in rehabilitation practice. Since this is the first study that examines learning processes of functional, goal-directed tasks executed during multiple practice sessions with a prosthesis, we applied a broad range of outcome measures, including changes in performing functional tasks and changes in movement coordination. For the latter, changes in kinematics of the movement were examined, which is novel. Based on earlier studies the kinematic variables of primary focus will be reaching and grasping time, the plateau phase in the hand aperture that characterizes coordination of hand opening and hand closing in prehension with a prosthesis, fixation time, and joint angles [8,26,27]. Results could reveal in what way motor coordination improves to provide hints as where to focus on during a training program.

Second, it is important to know what types of tasks need to be practiced to optimize learning [28,29]. The tasks included in this study are based on the actions that are performed with a prosthesis during daily life: direct grasping, indirect grasping—handing over an object from the sound hand to the prosthetic hand—and fixating [30]. Each task was studied separately to be able to extract information concerning the learning processes for each task individually in three separate groups. The changes in performance per task can then be studied, which provides information about the best task to facilitate learning, while the combination of tasks in a fourth group resembled rehabilitation and daily life more closely.

The third aspect in this study concerns grip force control of the prosthetic hand. Modulating grasping forces with a prosthetic hand is a skilled dexterous activity that is not easily mastered, and a good level of grip force control is one of the highest goals in rehabilitation [31]. Good grip force control is very difficult for prosthesis users since most of the feedback— including proprioception and tactile sense—lacks in prostheses. Several studies have already shown that prosthesis users are able to improve grip force control despite the lack of feedback [5,32,33]. To examine the grip force control in this study, objects were used that differed in compliance and therefore required different amounts of grip force.

The main goal of the current study is to describe the changes in performance over time that take place while learning to use an upper limb prosthesis. The study was designed to answer the following questions: i) what are the changes in the movements over time; ii) how do the different types of tasks influence the learning process; and iii) do the participants learn to control grip force, and if so, how does this process develop throughout the learning sessions.

## Methods

### Participants

An experimental group (15 males and 16 females; mean age (sd) = 20.27 (2.35) years) and an age- and sex-matched control group (15 males, 16 females; mean age (sd) = 21.2 (2.18) years) participated in the study. All participants were able-bodied, had normal or corrected to normal vision, were right-handed, and had no earlier experience with a prosthetic simulator. For the learning sessions, the participants in the experimental group were randomly assigned to one of four learning groups. One group learned direct grasping (DG, N = 8), one group learned indirect grasping (IG, N = 8), one group learned fixating (FIX, N = 7), and one group learned a combination of all three tasks (COM, N = 8). The participants in the control group only performed two tests and did not practice in between. The study was approved by the local ethics committee (METc application NL26993.042.09) and an informed consent was signed before the start of the experiment. The participants received a gift voucher afterwards.

### Apparatus

**Figure 1:**
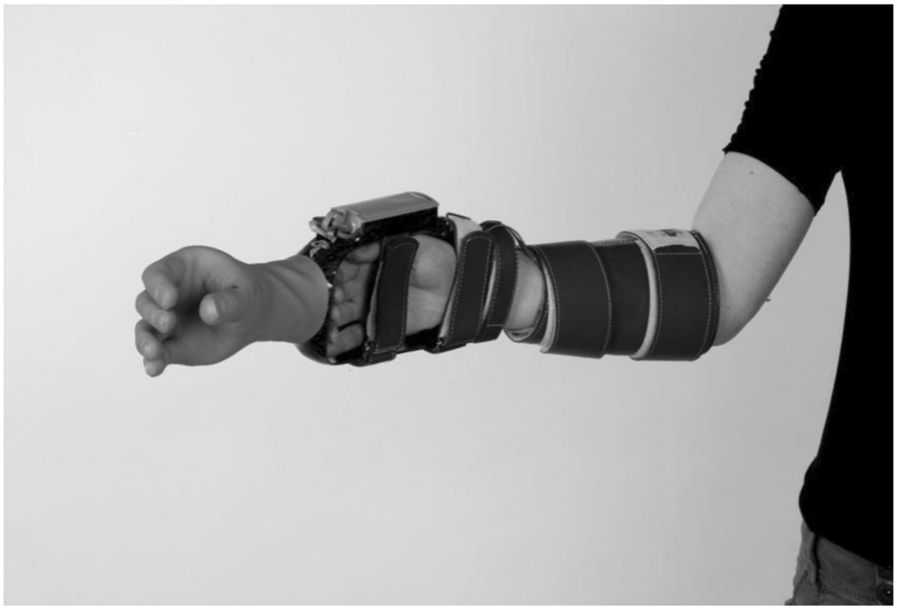
The myoelectric simulator.

The myoelectric simulator was developed to closely resemble a myoelectric forearm prosthesis (Figure 1), consisting of a myoelectric hand (MyoHand VariPlus Speed®, Otto Bock, with hand opening and hand closing speed between 15-300 mm/s and grip force control between 0 and 100 N). The height of the myoelectric signals was proportionally related to the hand opening as well as closing speed of the hand or the grip force. The hand was attached to an open cast in which the hand could be placed and a splint along the forearm. The splint was adjustable in length and was attached to the arm using a self-adhesive (Velcro) sleeve. The hand was controlled by changes in the electric muscle activity, detected by two electrodes that were placed on the forearm. Activation of extensors opened the hand whereas flexors closed the hand. The exact positions of the electrodes were determined after palpation of the most prominent contraction of the muscle bellies of the extensors and flexors. Subsequently, these locations were marked to place the electrodes. To check the correct position of the electrodes, the Prosthetists’ Assistant for Upper Limb Architecture (PAULA, Otto Bock®) was used to visualize the myoelectric signals, in conjunction with 757 M11 MyoBoy® connected to a PC. In this way the placement of the electrodes was such that the highest myoelectric signal was produced. The sensitivity of the electrodes was adjusted to the high level (66) as indicated by the MyoBoy and PAULA.

Three Optotrak 3020 systems (Northern Digital, Waterloo, Canada, sampling frequency 80 Hz) were used to record the positions of 30 infrared light emitting diodes (LEDs) attached to the trunk, the prosthetic arm, and the objects. One LED was placed on the ulnar border of the thumbnail, and one along the radial border of the nail of the index finger of the prosthetic hand. Four rigid bodies, triangles of hard PVC with a LED in each corner, were fixed according to Van Andel et al. [34]. One rigid body was placed laterally on the prosthetic wrist just proximal to where the radial and ulnar styloid would be, one on the upper arm just below the insertion of the deltoid muscle, one on the flat surface of the acromion, and one on the manubrium of the sternum. Two LEDs were placed on each of the objects used in the tasks.

A Bertec force plate (sized 40 cm × 60 cm, sampling frequency 300 Hz), synchronized with an Optotrak Data Acquisition Unit, was used to measure forces applied to the table surface in the fixation tasks. The force plate was placed on top of the table in front of the participant. The increased height was corrected by a wooden platform of the same height as the force plate, placed underneath the participants’ chair.

The Southampton Hand Assessment Procedure (SHAP, 35) was used during pretests, posttests, and retention tests to capture transfer of performance improvement in tasks other than learned. SHAP consists of 26 tasks: 12 abstract object tasks—6 lightweight and 6 heavyweight objects—and 14 activities of daily living (ADL) tasks, and evaluates functionality of the hand. Time scores of each task are transformed in an overall Index of Functionality score (IoF). The IoF is a score of hand function, a sound hand scores normally between 95 and 100; lower scores reflect decreased hand function [35].

**Figure 2:**
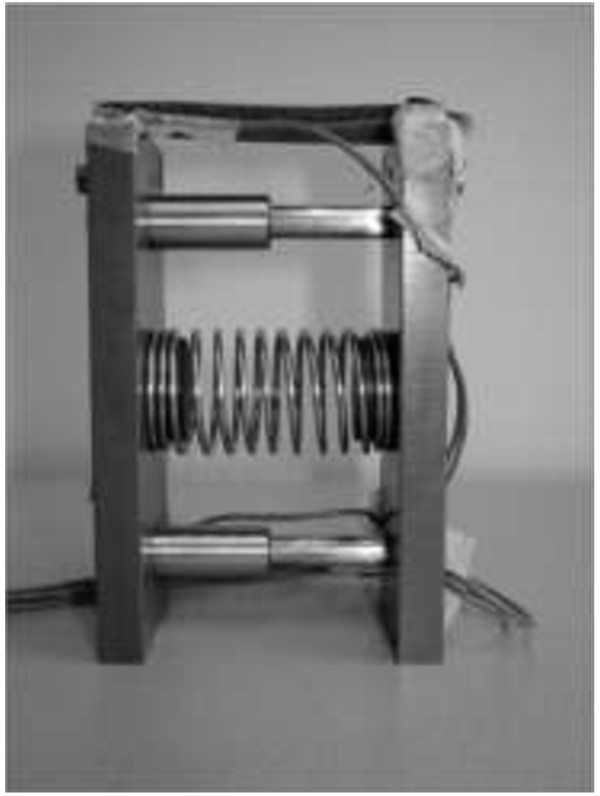
One of the deformable objects, consisting of two plates with a spring in between and Velcro mounted on top.

Three deformable objects and one solid object were used (6 cm × 3.5 cm × 9 cm) as objects in the grasping tasks. The deformable objects consisted of 2 plates with a spring between these plates (Figure 2). Each deformable object had a spring with a different resistance, requiring a different grip force before the object deformed—low-resistance object (LO; c = .17 N/mm); moderate-resistance object (MO; c = .57 N/mm); and high-resistance object (HO; c = 5.31 N/mm). The deformable objects simulated objects used in daily life, like a carton or a plastic cup. To simulate object manipulation—like opening the carton—a Velcro cover, mounted on top of the four objects, had to be pulled off from front to back of the object.

### Procedure and design

#### Tests

During the pretest prior to the learning sessions, SHAP was assessed to establish the baseline skill of the participants in both the experimental groups and the control group. After the last learning session, SHAP was administered again to determine the improvement of skills in the posttest. To determine the effect of learning over a longer period in the experimental group, two retention tests were assessed (see Table 1 for the experimental design). The control group only performed the first two SHAP tests, with the same time in between them as the pretest and posttest of the experimental group. This setup was chosen because SHAP is not validated yet for prosthesis users, and the control group served to examine the learning effect of performing SHAP twice.

**Table 1:** Set up of the experiment over the sessions

**Moments of measurement**	**Pretest**	**Session 1**	**Session 2**	**Session 3**	**Session 4**	**Session 5**	**Posttest**	**Retention test 2 weeks**	**Retention test 3 months**
Groups	DG	DG	DG	DG	DG	DG	DG	DG	DG
	IG	IG	IG	IG	IG	IG	IG	IG	IG
	FIX	FIX	FIX	FIX	FIX	FIX	FIX	FIX	FIX
	COM	COM	COM	COM	COM	COM	COM	COM	COM
	CO	-	-	-	-	-	CO	-	-

DG = Direct Grasping; IG = Indirect Grasping; FIX = Fixation; COM = Combination of DG, IG & FIX; CO = Control group.

Participants sat comfortably at a table, with their arms resting on the table and elbows in approximately 90 degrees, conform the SHAP manual. Prior to each task, task instructions were given. Different from the standardized SHAP protocol, the participants were not allowed to practice each task in advance to avoid premature learning during the pretest. The participants commenced each task with the prosthetic hand closed, and pressed a timer before and after executing each task for time measurement.

#### Sessions of the experimental group

During five sessions, spread out over a 2-week-period, participants learned the task(s) they had been assigned to (Table 1). Each session started with fitting of the prosthetic simulator, the LEDs and rigid bodies of the registration system. An Eyelink helmet (EyeLinkII, SR Research) was put on the head of the participants to measure gaze behavior of the participants. Prior to the start of the measurements, both Optotrak and Eyelink systems were calibrated. In this study, the results of the gaze data will not be reported, therefore we will not present details on that behalf.

For *direct grasping*, participants were instructed to pick up the object in front of them with the prosthetic hand, lift it, manipulate the object by pulling off the Velcro cover with the sound hand, and return it to the same position. The starting position of the prosthetic hand was located 15 cm from the edge of the table, and the object was located 30 cm distal from the initial hand position, both in line with the shoulder. During *indirect grasping*, the object was situated in the sound hand, and participants were instructed to hand over the object to the prosthetic hand, manipulate the object and return it to the starting position of the prosthetic hand. The initial positions of the sound and prosthetic hand were 25 cm from the edge of the table opposite to each other in the frontal plane, with 30 cm distance between both hands. The middle between the hands was aligned with the body midline. For both grasping tasks, participants had to execute the tasks as quickly but as accurately as possible, without deforming the objects.

Four different tasks were administered during *fixation*. Participants had to fixate i) a case with a flat design and zipper located at one side on top of the case, while unzipping and zipping the case with the sound hand; ii) a ruler on top of two dots—placed 20 cm horizontally from each other—with the prosthesis, while drawing a straight line between the dots with a pencil held in the sound hand; iii) a sharpener to sharpen a pencil by turning the handle of the sharpener 3 times with the sound hand; and iv) a piece of cloth to unbutton three buttons. The objects were placed on the force plate, 25 cm from the edge of the plate, aligned with the body midline. Participants were instructed to fixate the object with the prosthesis as still as possible during the task execution.

No further instructions were given for all three types of tasks (DG, IG, and FIX) to capture the natural developing changes in movement over time. The participants were informed that the spring stiffness’s of the three objects differed, the stiffness was also marked on the object, however, they were not allowed to practice with the objects beforehand. Each session contained 60 trials per group. The DG, IG, and FIX group performed 15 trials with each of the 4 objects in a random order, resulting in 60 trials per session. The COM group performed 5 trials per object and per task (DG, IG, and FIX), resulting in 20 trials per task and thus in 60 trials per session, with a randomized order of tasks (resulting in a blocked-repeated structure).

### Data analysis

#### Analysis of tests

Time scores of SHAP were entered into the SHAP website [36], which provided an overall Index of Functionality (IoF) score. Apart from the IoF, the time scores of the tasks were analyzed separately to obtain more detailed information. The time scores were transformed to z-scores, which are normalized scores with a mean of 0 and a standard deviation of 1, enabling comparison of all tasks. Z-scores were calculated by subtracting each score, thus for each participant and for each task over all tests, from the mean of all scores, and then dividing the resulting score by the standard deviation. Further, mean z-scores were calculated for each type of task in SHAP: abstract light, abstract heavy, and ADL. Two repeated measures ANOVA’s were executed on the mean z-scores; one to test the difference in performance between the experimental and control group with task type (abstract light, abstract heavy, and ADL) and test (pretest and posttest) as within-subject factors and group (experimental and control) as between subject factor; and the second to test the difference between tasks practiced in the sessions and the performance over a longer period, with task type (abstract light, abstract heavy, and ADL) and test (pretest, posttest, 2-weeks retention, and 3-months retention) as within-subject factors and group (DG, IG, FIX, COM) as between-subject factor. Three t-tests on the abstract light, abstract heavy, and ADL task types were executed on the pretest results to see whether the experimental group and the control group were equal in performance at baseline.

#### Analysis of the learning sessions data

**Figure 3:**
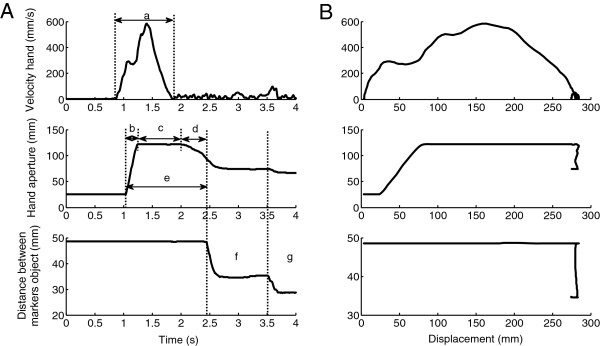
**Illustrative examples of a direct grasp trial with the low-resistance object.** Velocity of the hand, hand aperture, and object deformation are plotted against time **(A)** and against displacement of the hand from start position to the position of the object **(B)**. Dependent variables that are indicated in 4A: a = Reach time; b = Hand open phase; c = Plateau phase; d = Hand close phase; e = Total Grasp time; f = Compression during grasp; g = Compression during manipulation.

The onset and termination of the dependent variables of the end-point kinematics in the grasping tasks were determined using the Multiple Sources of Information method introduced by Schot et al. [37] (see Table 2) that was implemented in custom written Matlab programs. Reach time and peak velocity of the reach were determined for the transport phase. Hand opening time, plateau time, hand closing time, total grasp time (see also Figure 3), maximal aperture, mean velocity of hand opening, and mean velocity of hand closing were calculated for the grasp phase. Grasp was defined by the 3D distance between the markers on the thumb and index finger. Synchronization at end, which reflects the timing of the end of the reach and the grasp, was computed by dividing the time of grasp termination by the time of reach termination. A score of 1 stands for simultaneous ending of the reach and grasp. When the grasp ended later than the reach, scores exceeded 1, and when the grasp ended before the end of the reach, scores were below 1. Compression of the object was calculated by computing the 3D distances between the two markers on the opposite ends of the object, and determined for two moments: maximal compression during the initial grasp and maximal compression during manipulation of the object. The applied force during the initial grasp (Force at moment of grasp) and during manipulation (Force during manipulation) was subsequently derived from the constant of each of the springs: F(N) = constant of the spring (N/mm)* compression of the object (mm).

**Table 2:** The cut-off thresholds of the dependent variables for the end-point kinematics

**Variables**	**Description**	**DG**	**IG**
Start reach	X-position and Z-position of the hand on the table	90 < x-position hand < 150 mm	500 < x-position hand > 600 mm & z-position hand < 90 mm
	The hand is closed at the start	Aperture hand < 30 mm	Aperture hand < 30 mm
	Velocity of the hand starts to increase	10 < velocity hand < 50 mm/s	10 < velocity hand < 50 mm/s
End reach	The hand must be near the object	390 < x-position hand < 500 mm	0 < distance hand-object < 35 mm
	Velocity of the hand slows down	0 < velocity hand < 10 mm/s	0 < velocity hand < 20 mm/s
	Position of the object is not changed (only DG)	z-position object <87 mm	-
Start grasp	Aperture of the hand starts to increase	20 < aperture hand <50 mm	20 < aperture hand < 50 mm
	Velocity of hand opening starts to increase	Velocity hand opening > 20 mm/s	Velocity hand opening > 20 mm/s
End grasp	Aperture of the hand about size object	65 < aperture hand < 95 mm	65 < aperture hand < 95 mm
	Velocity of hand closing decreases to 0	0 < velocity hand closing < 15 mm/s	0 < velocity hand closing <15 mm/s
	Grasp has ended as object starts to move (only DG)	84 < z-position object < 100 mm	-
	The hand must be near the object	390 < x-position hand < 500 mm	0 < distance hand-object < 35 mm
Start plateau	Aperture is around maximum	90 < aperture hand < 150 mm	80 < aperture hand < 140
	Velocity of hand opening decreases to 0	0 < velocity hand opening < 20 mm/s	0 < velocity hand opening < 20mm/s
	Position of object is not changed yet (only DG)	z-position object < 87 mm	-
End plateau	Aperture is around maximum	90 < aperture hand < 150 mm	80 < aperture hand < 140 mm
	Velocity of hand closing starts to increase	15 < velocity hand closing < 30 mm/s	15 < velocity hand closing < 80 mm/s
	Position of object is not changed yet (only DG)	z-position object < 87 mm	-

DG: Direct Grasping; IG: Indirect Grasping; Mm: millimeter; s: second.

The force data of the fixation tasks, sampled by the force plate, was processed using custom made Matlab programs. The force perpendicular to the force plate (Fz) was used to determine maximal Fixation force during a trial. Fixation time was determined as the time that the applied force exceeded a threshold of 2 N.

Joint angles were calculated following the recommendations of the International Society of Biomechanics (ISB) proposed by Wu et al. [38]; see also [34,39]. The following angles were analyzed: flexion-extension, lateral bend, and rotation of the trunk; plane of elevation, elevation, and internal-external rotation of the shoulder; and elbow flexion-extension. Note that plane of elevation and elevation of the shoulder both determine the angle between the upper arm and trunk. Only the above mentioned trunk, shoulder and elbow angles at the side on which the prosthetic simulator was attached, were determined. Time of each movement was normalized (0-100%) to facilitate comparison. Range of Motion (ROM) for each angle was calculated by subtracting the minimum value from the maximum value of the angle in each trial.

The data were processed using Matlab (The Mathworks Inc, MA, USA). Trials were rejected when markers were obscured so that one or more of the above mentioned variables could not be determined. Repeated measures ANOVA’s were applied on each of the dependent variables (reach time, hand opening time, plateau time, hand closing time, total grasp time, mean velocity of hand opening, mean velocity of hand closing, synchrony at end, compression at moment of grasp, compression during manipulation, force at moment of grasp, force during manipulation, fixation force, and fixation time) with session (session 1 to session 5) and object (LO, MO, HO, and solid for the grasping tasks; and case, sharpener, buttons and ruler for the fixation tasks) as within-subject factors and group as between-subject factor. When sphericity was violated, the degrees of freedom were adjusted with the Greenhouse-Geisser correction. An α of .01 was used because of the large number of analyses performed. Post hoc tests on main effects used Bonferroni corrections. Generalized eta-squared [40] was used to calculate effect sizes, and interpreted according to Cohen’s recommendation [41] of .02 for a small effect, .13 for a medium effect, and .26 for a large effect. Only effects of .02 and larger are discussed in the results.

## Results

### Tests

**Figure 4:**
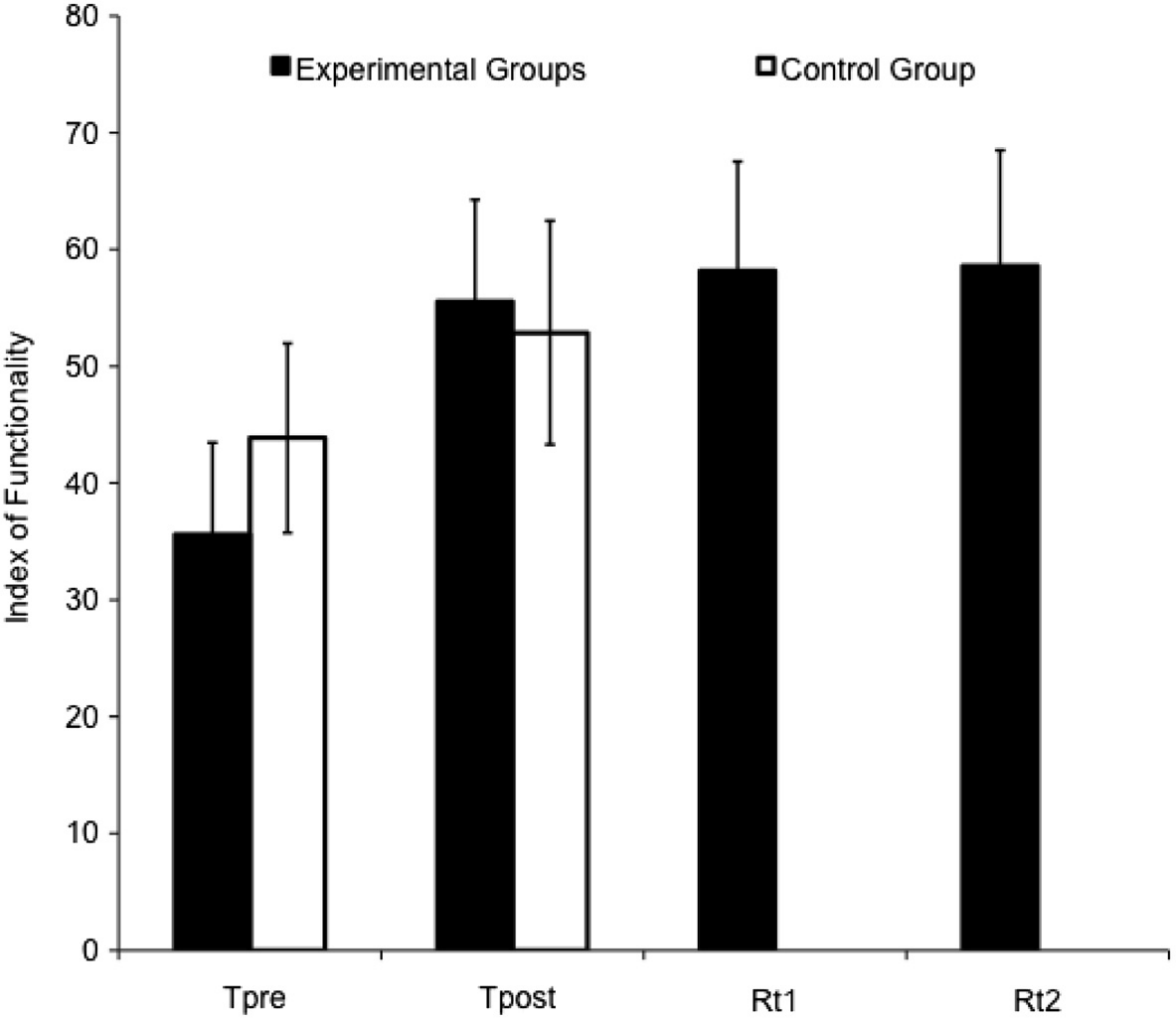
Mean (± SD) Index of functionality scores on SHAP for the experimental and the control groups on the different test times: pretest (Tpre), posttest (Tpost), retention test 1 (Rt1) and retention test 2 (Rt2).

The participants in the experimental group improved from a mean Index of Functionality (IoF) score of 35.61 on the pretest to 55.52 in the posttest (Figure 4). The performance remained on the same level during the retention tests, with a IoF score of 58.16 on retention test 1 and 58.58 on retention test 2. The control group improved as well from the first to the second test (mean = 43.87 and 52.87, respectively; see Figure 4). Three t-tests confirmed that the control group and the experimental group did not differ significantly from each other at baseline (t = .22, p = .83 for the abstract light tasks; t = -.50, p = .62 for the abstract heavy tasks; and t = 1.61, p = .11 for the ADL tasks).

Although the ANOVA on the z-scores showed that both the experimental group and the control group improved on SHAP (F_(1,59)_ = 153.18; p = .00; η_G_^2^ = .33), an interaction-effect of test by group revealed that the experimental group improved significantly more on the posttest compared to the control group (F_(1,59)_ = 21.61; p = .00; η_G_^2^ = .07).

A large effect of test (F_(1.37, 37.02)_ = 93.19; p = .00; η_G_^2^ = .49) showed that, within the experimental group, participants improved significantly on both the posttest and the retention tests compared to the pretest (p’s = .00 in pairwise comparison). The participants improved most on the light-weight abstract tasks over the time, revealed by a small interaction-effect of test by task (F_(2.64, 71.40)_ = 4.75; p = .01; η_G_^2^ = .03). The four experimental learning groups did not differ significantly from each other.

### Learning sessions

#### Grasping tasks - kinematics and applied grip force

Figure 3 shows a typical profile of the performance during a direct grasping trial. During the approach phase, the hand reaches towards the object. In the reach the hand opens to a maximal hand aperture, stays at a plateau for a while, and starts to close when the hand is near the object. During the grasp phase the object is picked up, and two types of compression of the object can be determined. The first compression occurs immediately when the object is picked up, and the second—further— compression occurs when the Velcro strip is pulled off.

Table 3 provides an overview of the mean (M) and the standard error (SE) of all significant main effects with an effect size of ≥ .02. A main effect of session shows the means of each of the five sessions, calculated over the objects and over the groups, while a main effect of object shows the means of each of the objects, calculated over the sessions and over the groups.

**Table 3:** Significant main effects in the learning sessions with an effect size of ≥ .02

	**Dependent variable**	**Within/between subject factor**		**Mean (SE)**	**95% CI lower–upper**	**F**	***p***	**η**_**G**_^**2**^
Reaching tasks: direct grasping and indirect grasping	Reach time (s)	Session	1	1.49 (.07)	1.36–1.63	5.66	.00	.03
			2	1.36 (.05)	1.25–1.47			
			3	1.33 (.05)	1.23–1.44			
			4	1.36 (.05)	1.26–1.47			
			5	1.35 (.06)	1.23–1.47			
	Plateau time (s)	Session	1	0.93 (.06)	0.82–1.04	10.43	.00	.05
			2	0.75 (.05)	0.65–0.85			
			3	0.72 (.04)	0.64–0.81			
			4	0.78 (.05)	0.68–0.88			
			5	0.78 (.05)	0.69–0.88			
		Object	LO	0.84 (.04)	0.76–0.93	11.66	.00	.02
			MO	0.83 (.05)	0.73–0.93			
			HO	0.77 (.04)	0.69–0.86			
			Solid	0.73 (.05)	0.63–0.82			
	Hand close time (s)	Object	LO	0.79 (.06)	0.68–0.91	35.72	.00	.11
			MO	0.73 (.05)	0.62–0.83			
			HO	0.57 (.05)	0.48–0.69			
			Solid	0.49 (.04)	0.41–0.57			
	Total grasp time (s)	Session	1	1.98 (.11)	1.75–2.21	8.66	.00	.03
			2	1.72 (.10)	1.51–1.92			
			3	1.67 (.08)	1.51–1.83			
			4	1.77 (.09)	1.59–1.95			
			5	1.77 (.09)	1.58–1.95			
		Object	LO	1.99 (.09)	1.79–2.18	32.54	.00	.07
			MO	1.89 (.09)	1.69–2.09			
			HO	1.68 (.09)	1.50–1.86			
			Solid	1.56 (.09)	1.38–1.74			
	Mean closing velocity (mm/s)	Object	LO	84.95 (5.87)	72.95–96.95	13.48	.01	.04
			MO	86.02 (6.25)	73.24–98.80			
			HO	86.64 (6.40)	73.55–99.73			
			Solid	109.42 (8.81)	91.40–127.44			
	Synchrony at end	Object	LO	1.55 (.04)	1.46–1.64	20.19	.00	.08
			MO	1.51 (.04)	1.42–1.59			
			HO	1.43 (.04)	1.35–1.51			
			Solid	1.34 (.03)	1.27–1.41			
	Compression at moment of grasp (mm)	Object	LO	10.09 (.84)	8.36–11.82	131.35	.00	.47
			MO	10.20 (.62)	8.93–11.47			
			HO	1.38 (.21)	0.96–1.81			
	Compression during manipulation (mm)	Object	LO	13.22 (.98)	11.21–15.22	166.78	.00	.54
			MO	12.53 (.63)	11.25–13.82			
			HO	1.74 (.23)	1.27–2.21			
	Force at moment of grasp (N)	Object	LO	1.73 (.15)	1.43–2.02	27.12	.00	.19
			MO	5.81 (.35)	5.09–6.54			
			HO	7.34 (1.09)	5.11–9.58			
	Force during manipulation (N)	Object	LO	2.26 (.17)	1.92–2.61	32.67	.00	.22
			MO	7.14 (.36)	6.41–7.88			
			HO	9.21 (1.22)	6.72–11.70			
Fixation tasks	Fixation force (N)	Object	Case	41.33 (3.45)	33.14–49.52	25.31	.00	.53
			Sharpener	45.32 (3.20)	37.21–52.85			
			Buttons	30.22 (4.35)	19.58–40.85			
			Ruler	19.80 (1.82)	15.36–24.25			
	Fixation time (s)	Object	Case	4.97 (.67)	3.34–6.60	15.18	.00	.28
			Sharpener	5.57 (.87)	3.43–7.71			
			Buttons	6.83 (.50)	5.62–8.04			
			Ruler	9.06 (.90)	6.87–11.25			

SE: standard error of the mean; 95% CI lower–upper: 95% Confidence interval with lower and upper bounds; s: second; mm: millimeter; N: Newton; LO: low–resistance object; MO: moderate–resistance object; HO: high–resistance object; Solid: solid object.

During the five sessions, a decrease was seen in the reach time, the plateau time, and the total grasping time, mainly on the first three sessions (Table 3). Moreover, although not significant, a gradual decrease throughout the five sessions was seen in the amount of compression of the object and therefore in the amount of grip force applied during grasping (M (SE) for session 1: 4.68 (.38); session 2: 4.29 (.24); session 3: 4.08 (.21); session 4 (3.24 (.17); and session 5 3.12 (.14)) and manipulation (M (SE) for session 1: 5.60 (.45); session 2: 4.95 (.25); session 3: 5.03 (.24); session 4: 4.12 (.19); and session 5: 3.87 (.16)), which did not show leveling off. No significant main effect of group was found.

**Figure 5:**
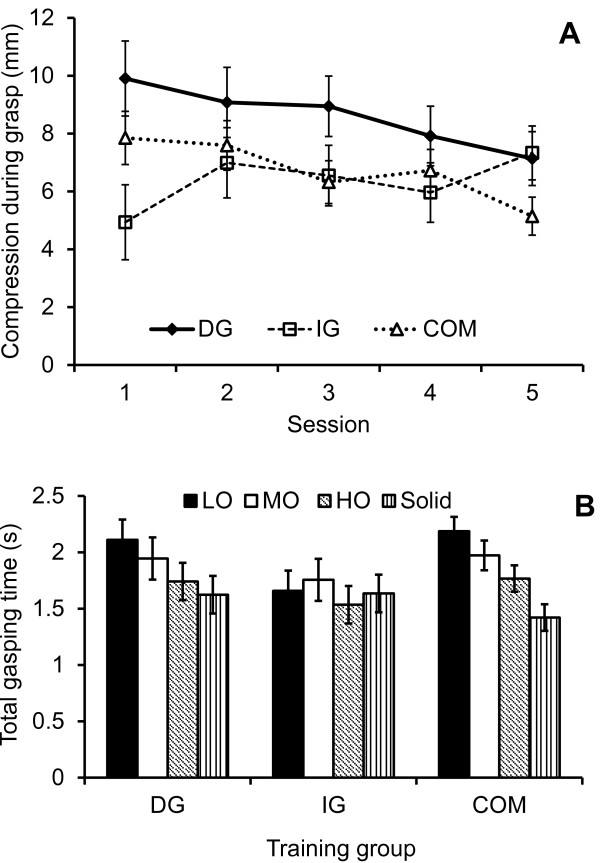
**Behavior of the different training groups during the grasping tasks. A)** The amount of compression of the objects over the sessions for each of the training groups that trained grasping (DG, IG, and COM); **B)** Total grasping time for each of the training groups for the different object resistances LO, MO, HO, and solid.

An interaction effect of session by group in the compression during grasp (F_(4.8, 69.9)_ = 3.22; p = .01; η_G_^2^ = .03) revealed that both the DG and COM group compressed the objects less over the sessions, while the IG group did not show this decrease in compression (Figure 5A).

With a low resistance of the object, thus with the object that was easier to compress, the plateau time, the hand closing time, and the total grasp time increased, whereas synchronization of the end of the reach and grasp and the mean velocity of hand closing decreased. The objects with low resistance resulted in larger compressions during grasp and manipulation of the object compared to the HO, while force production was less with the lower object resistances (Table 3).

Small interaction effects of group by object in hand closing time (F_(3.4, 49.9)_ = 6.27; p = .00; η_G_^2^ = .04), total grasp time (F_(4.2, 61.3)_ = 7.63; p = .00; η_G_^2^ = .04), and nearly significant synchrony at end (p = .03) revealed that a higher object stiffness resulted in a faster performance in the DG and COM groups, while the performance of the IG was about equal for the four objects (Figure 5B). Note that the performance of the IG group was overall faster than the other two groups. Nearly significant interaction effects of group by object for compression during grasping (p = .04) and compression during manipulation (p = .02) revealed that overall, the groups adjusted the performance to the characteristics of the objects, however, the IG group compressed the LO somewhat less than the DG and COM groups. The mean velocity of hand closing increased over increasing object stiffness (F_(3.3, 48.4)_ = 4.43; p = .01; η_G_^2^ = .03), where the IG group showed the overall fastest velocities for the LO, MO, and HO objects, while the COM group closed the hands the fastest for the solid object.

#### Fixation tasks – applied fixation force

The maximal fixation force used differed largely per object (Table 3), indicating that participants could adjust the fixation force as needed to finish the task. A small interaction effect of session by object (F_(12,72)_ = 3,16; p = .01; η_G_^2^ = .03) revealed a different fixation performance over the five training sessions, with slightly increasing maximal fixation force for the case, sharpener, and ruler over the sessions, whereas the maximal fixation force slightly decreased for the buttons task.

Although it did not reach significance, the fixation time decreased over the sessions of practice (p = .03; mean session 1: 8.78, session 2: 6.42, session 3: 6.43, session 4: 5.85, session 5: 5.55), and the time needed to fixate the objects differed largely (Table 3). Participants performed the case task the quickest, followed by the sharpener and the buttons, and the ruler task took most time. No differences were found between the FIX and COM group.

#### Joint angles in grasping and fixation tasks

**Figure 6:**
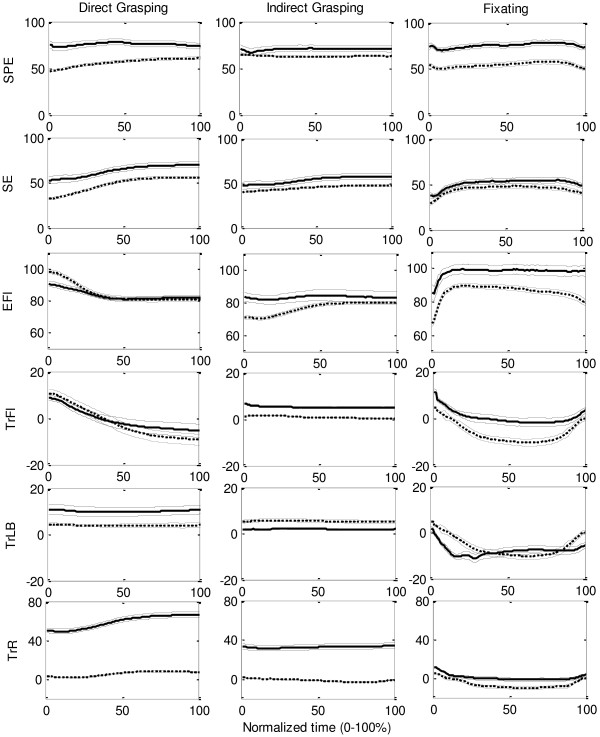
**The mean course in angles (degrees) of shoulder plane of elevation (SPE), shoulder elevation (SE), elbow flexion (EFl), thorax flexion (TrFl), thorax lateral bend (TrLB) and thorax rotation (TrR) from movement start to end in normalized time for the three types of tasks (direct grasping, indirect grasping, and fixating).** The solid lines represent the mean and standard error of the angles on the first session, the dashed lines represent mean and standard error for the fifth session.

The mean range of motion (ROM) and the standard deviation of the ROM of the shoulder, elbow, and thorax decreased mainly from the first to the second session. Figure 6 shows the angles of the shoulder, elbow, and thorax on the first and the fifth session. Overall, the ROMs were the highest for the fixation tasks, and the lowest for the IG task. The fixation tasks required the highest abduction angles—reflected by the lowest degree in plane elevation where 0° is abduction and 90° is forward flexion of the arm. All tasks were performed with the thorax in some forward flexion, lateral flexion to the left—away from the prosthesis side—and some left rotation. Over the sessions the lateral flexion and rotation of the thorax decreased to zero.

## Discussion

### Improvement over practice time

All groups improved on SHAP in the posttest, with a significantly larger improvement in the experimental groups compared to the controls. This implies that practicing with the prosthetic simulator improved overall performance, hence, not only familiarization to the task as the control group experienced but training is necessary to increase skills in prosthesis use. Moreover, the performance did not deteriorate in the retention tests. This is interesting, as it shows that the improvement is lasting, even after a period of non-use of the prosthesis. For movement times in the end-effector kinematics, fixation time, and range of motion, a fast improvement was seen between the first and second session, after which the improvement decreased over the next sessions and leveled off after three sessions. The learning process of the force control proceeded differently. Although not significant, the improvement in performance over the five sessions demonstrated an ongoing improvement in the learning process without leveling-off. These results make clear that controlling the hand, especially the fine-tuning of adjusting the opening and closing to different object characteristics, which reflect fine motor control [42], takes longer to learn than the gross motor control such as the positioning of the prosthetic arm in the surrounding space. This is not surprising, however, if one recalls that the joints and muscles around the shoulder and elbow are still intact and also used for these gross motor actions when using a forearm prosthesis. Therefore it is likely that, as also suggested by Metzger et al. [5], the existing sensory feedback in shoulder and elbow provided enough information to learn to control such movements quickly. On the other hand, the prosthetic hand has replaced the own hand, and needs to be controlled with the muscles that first mainly controlled the wrist instead of the hand. It is reasonable to presume that this results in a longer period to master control of the hand.

### Differences between experimental groups

During the learning sessions, the different tasks led to a difference in performance of the groups. Whereas the DG and COM improved over the sessions and adjusted the control of the hand to the characteristics of the objects, this was not seen in the IG group. Notice, however, that the IG group started off better and had an overall better performance; they were overall faster than the other two groups, and compressed the object with the low resistance less. The difference in performance could be explained in several ways. One of the reasons might be that during the IG task, more information can be retrieved about the deformable objects because of the involvement of the sound hand. This bimanual component in the indirect grasping included proprioception of the sound hand, which could have led to a better translation to the control signals of the prosthetic hand. Moreover, the participants were able to position the object with the sound hand into the prosthetic hand. Therefore, unlike the DG task, no attention had to be paid to positioning of the prosthetic hand with regard to the position of the object. Finally, the absence of improvement in IG group over sessions could also have resulted from their relatively good start, which might have left no room for improvement. This finding is important, since amputees need to achieve success when they start practicing with a prosthesis, in order to motivate them to continue practicing and to use the prosthesis. Therefore, we recommend starting with an IG task.

Even though the number of repetitions during practicing each individual task was less, the level of performance of the COM group in the functional test was equal to the other groups. Hence, less practice of each task in this group led to comparable results, which means that they have learned more in fewer repetitions. The advantage of the COM group was that they were able to use the information obtained during IG while performing DG, which might have helped to improve overall performance. Together with the blocked-repeated order of tasks in which they learned, these results could suggest that this particular structure of learning might lead to the best overall performance over time. Learning in a random manner—with several tasks learned at the same time—has been shown to lead to the best transfer of skills to other tasks than learned [43,44]. The blocked-repeated fashion that is used in this study has been suggested as the best training design to achieve the best overall performance [45,46]. This allows learning a task quickly while practicing it in a blocked order for several repetitions, whereas the repetition of these blocks would promote transfer of the skills.

The fact that all experimental groups performed equally on the SHAP tests after training is a finding that deserves attention. Especially the performance of the fixation group is remarkable. These participants only fixated objects during the learning sessions and did not learn to control the prosthetic hand actively, while SHAP tasks require active control of the hand. At the moment, we cannot provide a conclusive explanation for this lack of difference between the groups. What was noticed during the sessions, however, was that the prosthetic hand was often—unintentionally—opened during fixation, and participants had to close the hand again in order to start the next trial. This could imply that they did practice active control of the hand to some extent, and were therefore able to perform the SHAP tasks that required active control of the hand. The equal performances of the four training groups could suggest that experience and practice with the prosthesis in itself could provide enough training, however, we might expect that with longer training that has more specific feedback about hand control, differences between the groups could emerge.

### Improvement in grip force control

The participants were able to learn force control over practice sessions, with a gradual learning process that we expect to have continued when we had measured over an even longer period of time. The results demonstrate that with a prosthetic hand control of grasping force takes a long time, implying that it needs special attention and training to avoid crushing objects [47].

The fact that force control can be learned with a prosthetic hand has been reported earlier [48-51], however, this study is the first using compliant objects during goal-directed grasping tasks over a period of time, providing supplementary information on prosthesis control where other studies have only used rigid setups or non-goal-directed functional tasks to measure force control. It is surprising though, that most of the prehension research and control of the hand—both with sound hands and prosthetic hands—has been performed with rigid objects [52], since many objects are deformable in daily life. Interesting from the rehabilitation perspective is the fact that participants, relying solely on visual feedback because the prosthetic hand lacks the sensory information that is present in sound hands, were able to learn to control the force applied by the prosthetic hand. Thus, the still existing visual feedback provided enough information to learn force control to a certain extent. Since feedback plays a central role in motor learning [21,22], it is of interest to explore the role of feedback during the learning processes of learning to use a prosthesis further. Moreover, it is important to examine the relevance of providing augmented feedback such as visual, auditory, vibrotactile or verbal feedback see [53-56] for an overview of studies on augmented feedback during learning, especially while using the prosthesis handling compliant objects.

### Understanding underlying motor learning processes

A question that arises from this study is whether our results could provide insight in the understanding of motor learning and motor control. The results of the current study exposed the changes in performance over time. Moreover, the results indicate that there are different processes involved when learning to use a prosthesis, shown by the results on the different outcome measures that were analyzed. One of the approaches to motor learning that could be applied to these results is the dynamical systems theory [56]. The dynamical systems approach examines changes in the movement organization—and thus in performance—and the interaction between the learner and the environment at multiple levels of analysis that each have their own changing time scales [56,57]. The learner self-assembles the information that is available to learn organizing the movements to achieve the desired outcome [56,57]. It seems that without having proprioceptive feedback from the prosthetic hand, the remaining information was sufficient to learn movements to a certain extent. Gross positioning could be learned rather well because of the information that is left in the remaining arm, while fine control takes more time, possibly because of the reduced information that is available, as the learners could only exploit visual feedback. This is reflected in the different time scales of learning observed in the study. The learning curve observed in the gross movements—which is similar to curves found in most learning studies, c.f. Newell [56]—is faster than the fine control learning curve, which seems to have another, slower changing time scale. The dynamical systems approach could be an interesting approach to model different learning processes and the performance of a prosthesis user to be able to predict changes in future learning.

The dynamical systems approach to motor control is inspired by the work of Bernstein [58] who described the process of skill acquisition as learning to control the various degrees of freedom of the body. A human has many degrees of freedom of movement, and although there are less when using a prosthesis there are still abundant possibilities to achieve a desired outcome. One of the core questions in motor learning is how a learner finds the optimal solutions to achieve a certain goal [59]. The process of finding the optimal solutions has been examined by studying the origins of variability over learning, which is a characteristic that is reported in many motor learning studies [23,60-62]. While the current study was set up to get a global picture of the changes in performance over time, a next step would be to take a more closer look at these processes of change of the different learning curves, by examining the variability of performance over practice. Applying a method such as the Uncontrolled Manifold [63,64] or the tolerance-noise-covariation (TNC) method [60,62] would be very informative to use, since it decomposes variability into several components, which will provide more detailed insight in how to promote learning the most.

### Study limitations

The participation of able-bodied individuals using prosthetic simulators instead of amputees using real prostheses is a limitation of the study. The reason that we chose for this set-up is that there are only a very limited number of novice prosthesis users, and by studying able-bodied participants with a simulator many more subjects could be included. Moreover, it would not be ethical in this stage to deny novice prosthesis users the regular occupational therapy to be able to study the learning process in this set-up. Furthermore, comparing the current results to previous results provides indications that the use of the simulators are comparable to real prosthesis use in terms of SHAP scores [65,66] and kinematic profiles [8,26,27,66]. Therefore, the use of simulators seems to be justified. Another limitation might be that the control group was assessed only twice, during the pretest and posttest, but not during retention tests. We chose for this design because SHAP is not validated yet for prosthesis use and we deviated from the standard SHAP protocol. With hindsight, it might have been more appropriate to have measured the control group during retention tests as well.

Another factor that could be included in future research is the amount of mental effort that is required when learning to use a prosthesis. In the first part of the rehabilitation process a great amount of mental effort is required to learn to control the prosthesis. We expect that over learning the amount of mental effort will decrease, especially with the suggestions for clinical practice that emerged from this research. When mental effort is included in the outcome measures as well it would enhance our understanding further about how people learn to use their prosthesis, and in addition it might help us to determine the level of functioning of a prosthesis user during rehabilitation. This might be particularly true for learning grip force control because it takes a long time. Moreover, although we showed that grip force control can be learned it is unclear how these skills transfer to objects of different stiffness than practiced. It might be that mental load increases relatively a lot when objects of different stiffness need to be picked up. Future research is required to establish this because this is not explicitly tested in SHAP.

### Clinical application

The set-up of this study approaches a rehabilitation setting more than a single time measurement design, which leads to useful clinical insights. First, when designing an evidence-based training, more time should be spend on force control compared to gross movements with prosthesis, since learning grip force control requires much more time and attention. Second, patients should start to train with at least an indirect grasping task, thereby increasing the amount of useful information provided by the sound hand to perform the task. This information can then be used for other tasks as well, as seen in the COM group. Third, patients should train in a blocked-repeated fashion, allowing quick learning of a specific task in one block, and promoting transfer of skills by variable repetition of the blocks as well.

## Conclusion

Learning processes were examined in participants that learned to use a prosthetic simulator in different goal-directed tasks. Results showed that grasping force control took longer to learn than positioning of the prosthesis and that indirect grasping was beneficial for controlling the grip force. Practicing different tasks improved grasping control to the same level than training just grasping while the number of grasping trials in practice were less. Improvement in performance lasted even after a period of non-use. Suggestions for clinical practice are to focus specifically on grip force control of the hand, to start to train with an indirect grasping task, and to train in a blocked-repeated fashion.

## Abbreviations

ADL: Activities of daily living; ANOVA: Analysis of variance; COM: Combination group; DG: Direct grasping group; F: Force (N); FIX: Fixating group; HO: High-resistance object; IG: Indirect grasping group; IoF: Index of functionality; LED: Light emitting diode; LO: Low-resistance object; MO: Moderate-resistance object; ROM: Range of motion; SHAP: Southampton hand assessment procedure.

## Competing interests

This study was performed while Hanneke Bouwsema was financially supported by Otto Bock Healthcare GmbH, Vienna, Austria. By legal contract Otto Bock had no influence on the interpretation of the results and the wording in the manuscript.

## Authors’ contributions

HB participated in designing the study, conducted the experiment, performed the analyses, and drafted the manuscript. CS participated in designing the study and helped to draft the manuscript. RB participated in designing the study, participated in performing the analyses, and helped to draft the manuscript. All authors read and approved the final manuscript.
